# Supplementary figures and images for: Host- and microbial-mediated mucin degradation differentially shape Pseudomonas aeruginosa physiology and gene expression
Source: PLoS Pathog. 2025 Oct 3;21(10):e1013568. doi: 10.1371/journal.ppat.1013568 (PMC12503327; doi:10.1371/journal.ppat.1013568)

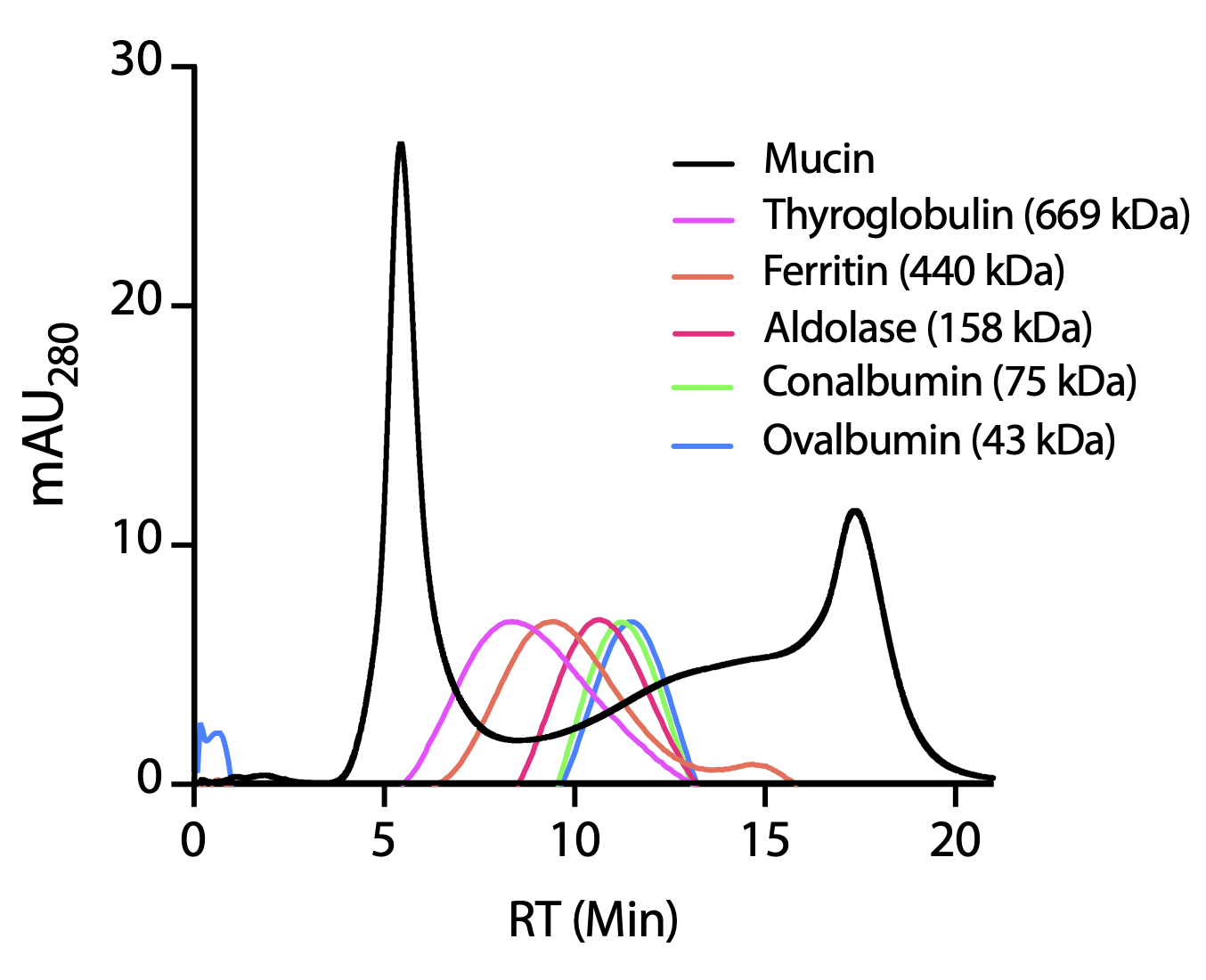

Supplement: S1 Fig — HMW standards in the GE Gel Filtration Calibration Kit as analyzed by FPLC. HMW compounds (i.e., intact mucin) elute earlier than those of lower molecular weight (i.e., degraded mucin). (TIF) [file ppat.1013568.s001.tif]

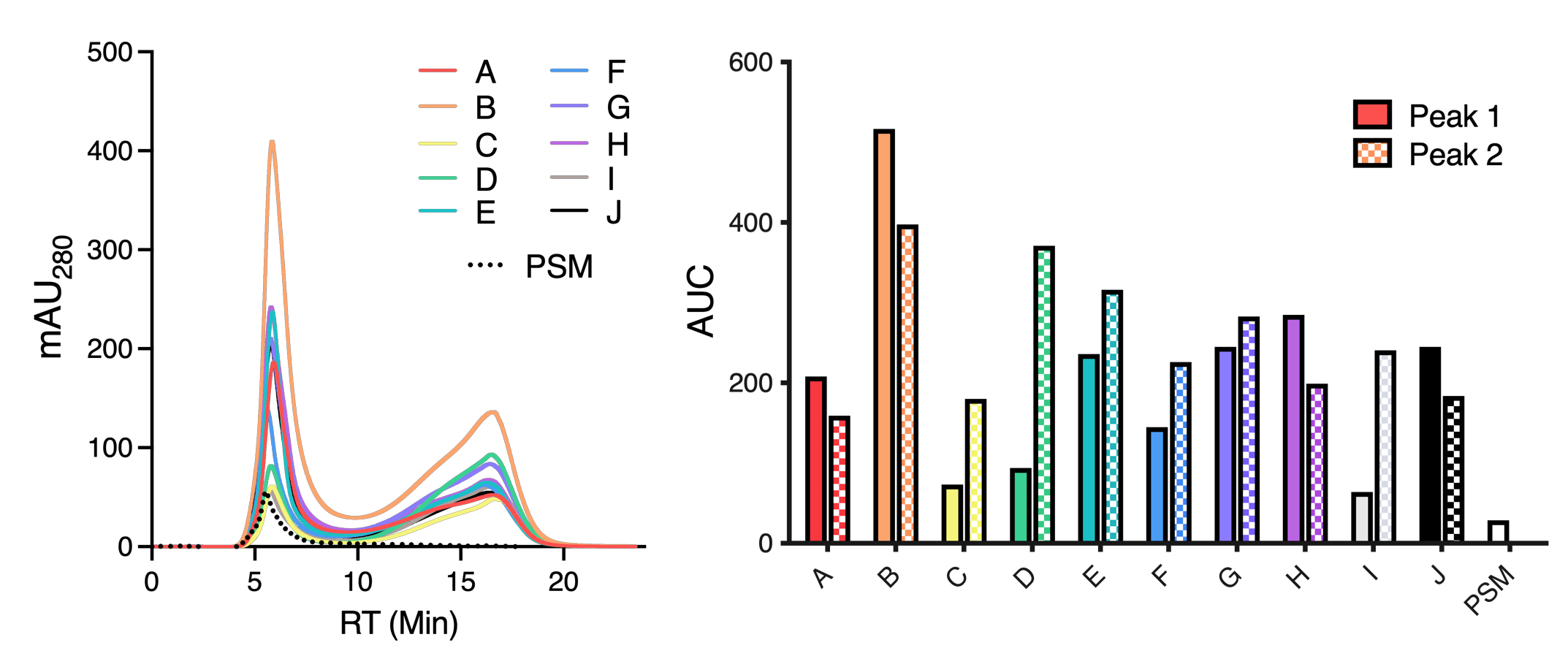

Supplement: S2 Fig — (a) Size exclusion chromatography profiles of mucins isolated from CF sputum relative to purified sinus mucin (PSM, dashed line). (b) Area-under-curve (AUC) data for Peak 1 (solid color) and Peak 2 (hatched color) of individual sputum samples compared to PSM. (TIF) [file ppat.1013568.s002.tif]

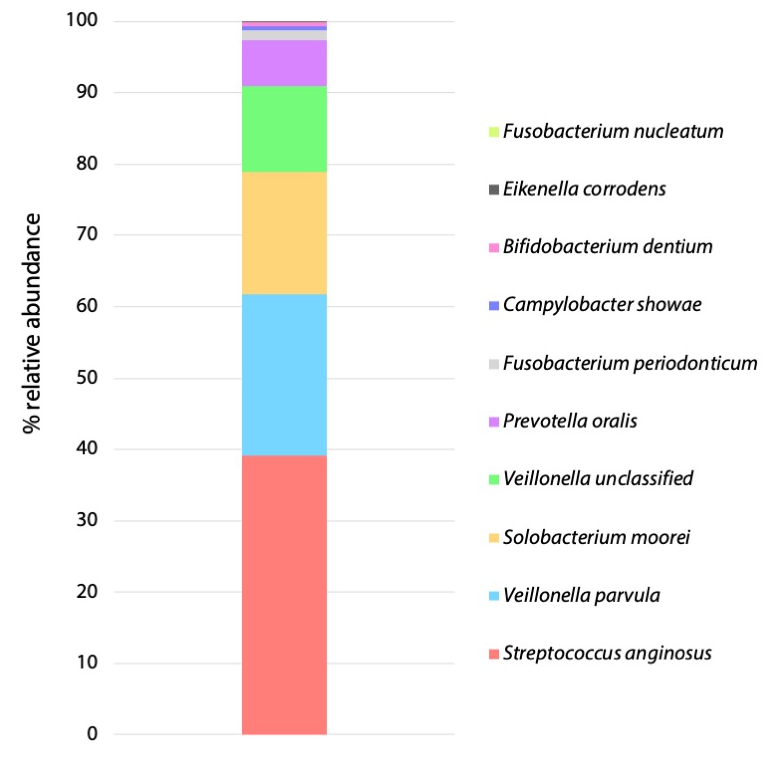

Supplement: S3 Fig — Composition of the anaerobic mucin degrading community (AMDC) as determined by 16S rRNA gene sequencing. (TIF) [file ppat.1013568.s003.tif]

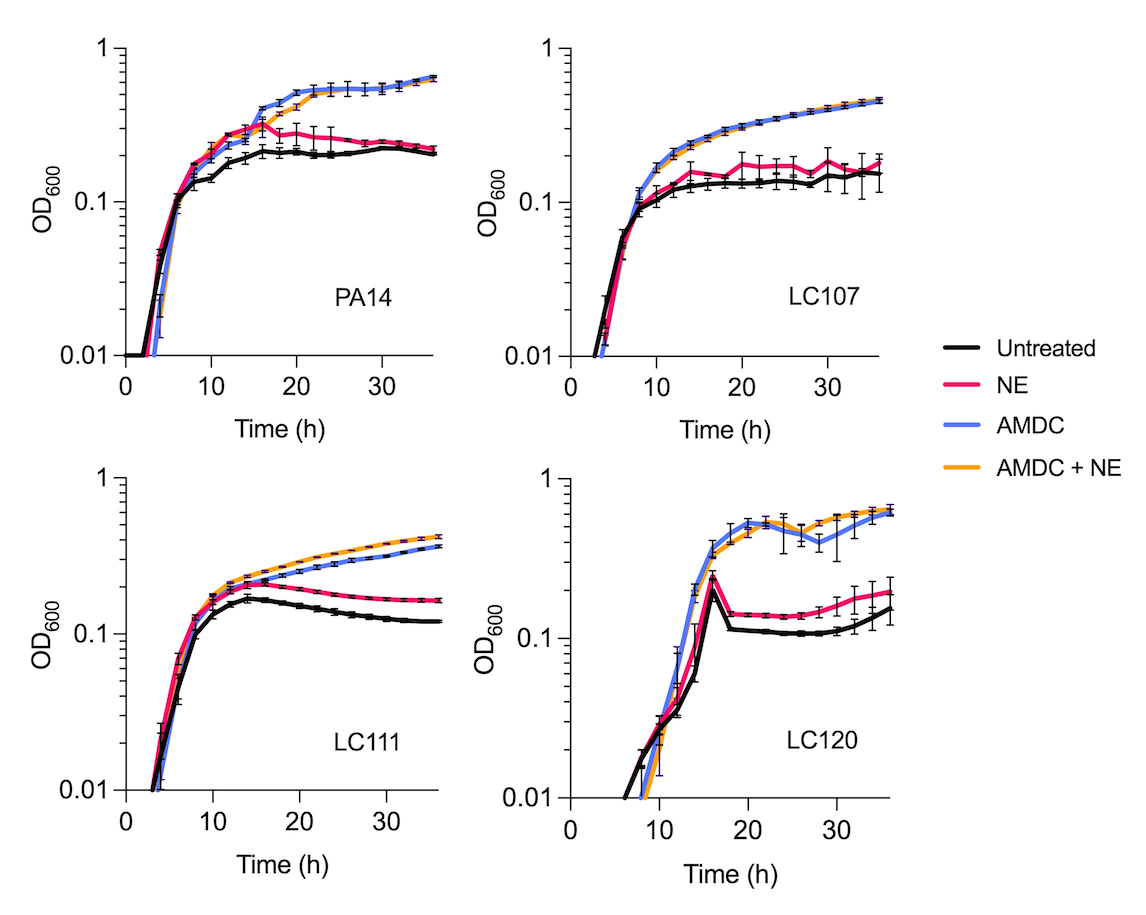

Supplement: S4 Fig — Growth curves of P. aeruginosa clinical isolates on untreated MMM, and NE-, AMDC-, and AMDC+NE-treated MMM. Each isolate exhibited increased growth in the cell-free supernatants of AMDC- and AMDC+NE-treated mucin. (TIF) [file ppat.1013568.s004.tif]
